# An Improved SEM Preparation Workflow for Plasma Membrane Imaging in HepG2 and IM-9 Cells

**DOI:** 10.3390/bioengineering13050541

**Published:** 2026-05-09

**Authors:** Laura Manin, Antonio Castelliti, Elena Stuppia, Filippo Laganà, Salvatore A. Pullano, Marta Greco

**Affiliations:** 1 Department of Health Sciences, Magna Graecia University of Catanzaro, 88100 Catanzaro, Italyantonio.castelliti@studenti.unicz.it (A.C.); elena.stuppia@studenti.unicz.it (E.S.); filippo.lagana@unicz.it (F.L.);; 2 Department of Electrical Engineering and Computer Science, University of Missouri, Columbia, MO 65211, USA

**Keywords:** scanning electron microscopy, HMDS, stepwise ethanol-to-HMDS substitution, plasma membrane preservation, cell surface morphology, drying artefacts

## Abstract

Accurate visualization of the plasma membrane is essential for assessing morphological changes induced by experimental perturbations. Scanning electron microscopy (SEM) enables high-resolution imaging of cell surface architecture, but the fixation, dehydration, and drying steps critically affect membrane preservation. Here, we present an improved SEM preparation workflow for two cultured cell models with different growth phenotypes, HepG2 and IM-9, and evaluate the effects of fixation time and drying strategy on plasma membrane preservation. Among the tested conditions, a shorter glutaraldehyde fixation time (15 min) combined with stepwise ethanol-to-HMDS substitution provided the best overall preservation of membrane continuity, cell shape, and surface regularity. Morphometric analysis supported the qualitative SEM observations, and HMDS-processed samples also showed better structural stability during storage under the tested conditions. This workflow provides a simple, reproducible, and cost-effective strategy for SEM-based analysis of cell surface morphology in cultured cell models.

## 1. Introduction

The preservation of plasma membrane integrity is crucial for morphological analysis and for the evaluation of membrane-associated structures [1,2,3]. Such visualization is highly relevant in both adherent and suspension cell systems, as the accurate assessment of membrane interactions and nanoscale surface alterations relies on the preservation of the plasma membrane ultrastructure [1,2,3,4]. Scanning electron microscopy (SEM) is a powerful tool for the high-resolution visualization of cellular ultrastructures, allowing a detailed observation of morphological features at the subcellular level, including the plasma membrane [5,6]. In addition to SEM-based ultrastructural imaging, complementary approaches for cellular characterization, including biosensing, computational modelling, and electrical-based techniques, have been increasingly explored to provide additional insight into cellular behaviour and function [7,8,9]. SEM imaging relies on a focused electron beam scanned across the sample in high vacuum conditions, generating secondary electron signals that provide nanometre-scale surface information [10]. The preparation of biological samples for SEM is critical to preserve their native morphology [11,12]. Key steps in SEM sample preparation include chemical fixation, buffer washing, graded dehydration and final drying, each contributing to the structural stabilization of the specimen [1,2,3]. Chemical fixation covalently stabilizes proteins and membrane components, preventing autolysis and preserving the native ultrastructure [10,11,12]. Subsequent washing removes unreacted fixative and normalizes the ionic environment before dehydration [11,12]. Graded ethanol dehydration progressively replaces intracellular water, minimizing osmotic shock and reducing the risk of membrane collapse. Once dehydrated, the sample must be fully dried, as any residual water or uncontrolled liquid–gas transitions in the SEM vacuum chamber can severely distort soft cellular surfaces [13,14]. SEM preparation protocols follow the same general sequence but differ in key parameters, including fixation time, fixative composition, dehydration strategy, and drying method, which can lead to variable membrane preservation across studies. Among drying approaches, critical point drying (CPD) remains the gold standard because the liquid–gas transition of CO_2_ eliminates surface-tension-induced deformation, although the method is expensive and time-consuming [15]. Hexamethyldisilazane (HMDS) is widely used as a chemical drying agent in SEM preparation and can provide good ultrastructural preservation, whereas air-drying often produces pronounced shrinkage, wrinkling and membrane deformation due to the high surface tension of evaporating ethanol [15,16,17]. Despite its widespread use, most HMDS protocols employ a direct transition from absolute ethanol to 100% HMDS, without stepwise substitution [18,19]. This abrupt solvent exchange may impose mechanical stress on the membrane and has been only limitedly evaluated in plasma-membrane-focused applications. Furthermore, existing protocols rarely examine fixation time as a variable, even though the extent of aldehyde crosslinking is known to influence membrane elasticity and fine structural integrity [15,18,19]. Therefore, this study aimed to compare selected glutaraldehyde fixation durations and drying strategies for high-fidelity SEM visualization of the plasma membrane while minimizing preparation artefacts in the tested cell models.

## 2. Materials and Methods

### 2.1. Cell Line

Human hepatocellular carcinoma HepG2 cells (ATCC HB-8065) and IM-9 human B-lymphoblastoid cells (ATCC CCL-159) were used as representative models with different growth phenotypes. This protocol requires standard human cell culture reagents (RPMI-1640 medium supplemented with heat-inactivated FBS, L-glutamine and penicillin/streptomycin) to ensure the optimal growth and viability of the HepG2 and IM-9 cells prior to fixation. RPMI-1640 was used for both cell lines to ensure consistent experimental conditions across adherent and suspension models, although EMEM is the recommended medium for HepG2 cells. RPMI-1640 is a nutrient-rich medium and has been reported to support the stable growth of HepG2 cells [20]. Cell growth, monolayer confluence, morphology, and viability were monitored before fixation, and only cultures meeting the stated viability threshold were processed for SEM preparation.

### 2.2. Sample Preparation for SEM

The protocol requires standard sterile consumables (2 mL microcentrifuge tubes, serological pipettes, tips, and 35 mm culture dishes pre-treated for adhesion). The samples were mounted on aluminum SEM stubs using conductive carbon tape. The centrifugation steps during fixation, ethanol dehydration, and HMDS processing were performed at 4 °C using a refrigerated centrifuge, while routine culture handling was carried out using a standard benchtop centrifuge at room temperature. A fume hood is needed for glutaraldehyde and HMDS manipulation. Cell morphology was checked using a phase-contrast microscope and final imaging was performed on a ZEISS EVO SEM controlled by SmartSEM software v.5.07.

The HepG2 cells were thawed from cryopreserved stocks and expanded to 60–70% confluence before harvesting for SEM preparation. The cells were cultured in RPMI-1640 medium supplemented with 10% (*v*/*v*) fetal bovine serum (FBS), 1% (*v*/*v*) L-glutamine and 1% (*v*/*v*) penicillin–streptomycin (P/S). The cells were maintained in plates at 37 °C in a humidified atmosphere containing 5% CO_2_ until reaching 60–70% monolayer confluence.

**Figure 1:**
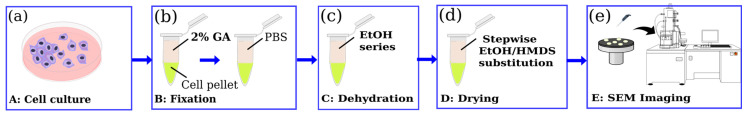
Proposed workflow for preparation of HepG2 and IM-9 cells for SEM imaging. (**a**) Cell culture and harvesting. (**b**) Fixation in 2% glutaraldehyde and PBS washing. (**c**) Dehydration in graded ethanol series (30%, 50%, 70%, 90%, and 100%). (**d**) HMDS drying using stepwise ethanol-to-HMDS substitution. (**e**) Final mounting on conductive carbon tape and SEM imaging.

After routine expansion, The cells at 60–70% confluence were detached using trypsin–EDTA solution (0.25% trypsin, 0.02% EDTA). Cell viability was confirmed by trypan blue exclusion (>90%), and only viable cells were used for SEM preparation (Figure 1a). IM-9 cells were cultured in suspension and harvested by gentle centrifugation (~110× *g*, 5 min). The resulting pellet was then processed using the same fixation, dehydration, and drying workflow applied to the HepG2 cells. A total of 3.6 × 10^6^ cells per sample was used as the standard input for SEM preparation in both cell lines.

After harvesting, the cells were pelleted, washed once with cold 0.1 M PBS (pH 7.4), and centrifuged at ~160× *g* for 5 min at 4 °C to remove residual medium. The cells were fixed by adding 1.5 mL of 2.0% (*v*/*v*) glutaraldehyde solution prepared from a 25% stock in 0.1 M PBS (pH 7.4). Fixation was performed at 4 °C on a gentle rotator to ensure uniform mixing and complete contact between the fixative and the cell suspension. The procedure was carried out under two experimental conditions:

Condition 1: fixation for 30 min;
Condition 2: fixation for 15 min.

These two fixation times were compared to assess the effect of fixation duration on membrane preservation. After fixation, the cells were washed twice with 1 mL of 0.1 M PBS (pH 7.4), with each washing step including centrifugation at ~160× *g* for 5 min at 4 °C, to ensure the complete removal of residual glutaraldehyde and to terminate the fixation process (Figure 1b).

Dehydration of the fixed cells was performed using a graded ethanol series (30%, 50%, 70%, 90%, and absolute ethanol) prepared by diluting absolute ethanol with deionized water at room temperature. The cells were sequentially incubated in 1 mL of each ethanol solution as follows:

1. 30% ethanol, 5 min;
2. 50% ethanol, 5 min;
3. 70% ethanol, 5 min;
4. 90% ethanol, 5 min;
5. Absolute ethanol, twice, 10 min each.

After each incubation, the cells were gently pelleted and the supernatant was carefully removed before the next ethanol step. This graded dehydration protocol ensured the gradual removal of water while minimizing osmotic stress, thereby preserving cell morphology and membrane integrity for SEM imaging (Figure 1c).

In the first experiment, the fixed and dehydrated cells were allowed to air-dry under ambient conditions for 30 min inside a laboratory hood to prevent contamination. This approach enabled complete drying prior to SEM analysis. In the second experiment, chemical drying was performed using hexamethyldisilazane (HMDS) to replace ethanol gradually and preserve cellular morphology (Figure 1d). The cells were sequentially incubated at room temperature, in 1.5 mL of each solution at 3:1, 1:1, and 1:2 (*v*/*v*) EtOH:HMDS ratios, followed by three incubations in 100% HMDS, with each step lasting 10 min. After each incubation, the cells were centrifuged at ~70× *g* for 5 min to remove the supernatant and ensure the complete replacement of the previous solution. Following the final HMDS incubation, the residual HMDS was allowed to evaporate slowly in an open tube under a chemical fume hood for 30 min, to prevent rapid drying and preserve membrane integrity for SEM imaging (Figure 1d).

### 2.3. SEM Imaging

The final preparation involved transferring the freshly dehydrated and dried cell suspensions onto a metal SEM stub covered with conductive carbon adhesive tape (Figure 1e). A 2–5 μL aliquot of the suspension was deposited onto the carbon tape using a micropipette and allowed to adhere before imaging. The mounted specimens were left under the hood to allow residual solvent evaporation and stable adhesion to the stub before SEM imaging. Once dried, the mounted samples were analyzed using a Scanning Electron Microscope (Carl Zeiss, Oberkochen, Germany, EVO HD15) equipped with SmartSEM v05.07 software, enabling morphological visualization of the plasma membrane. The samples were imaged without conductive coating at low accelerating voltage (5 kV) under variable-pressure conditions to minimize charging artefacts in biological specimens. Imaging parameters were adjusted to reduce beam-induced charge accumulation while preserving overall cell morphology. These imaging settings were applied consistently across the samples to minimize charging artefacts during uncoated, variable-pressure SEM analysis.

### 2.4. Morphometric Analysis and Statistical Evaluation

Morphometric analysis was performed using ImageJ v1.54s (Fiji, NIH, Bethesda, MD, USA). The SEM images were analyzed to extract individual cell contours for quantitative evaluation. For each condition, 15 cells were analyzed, sampled from four independent biological replicates (approximately 3–5 cells per replicate) and randomly selected from multiple fields of view to minimize selection bias. To avoid treating individual cells as independent biological replicates, cell-level measurements were averaged within each biological replicate before statistical testing; the reported cell number refers to descriptive cell-level morphometry. The following parameters were measured: projected cell area, circularity, solidity, and boundary irregularity index (BII). Circularity and solidity were calculated using standard ImageJ functions. The boundary irregularity index was calculated as:

(1) BII = (P − P_convex hull_)/P_convex hull_

where P represents the measured cell perimeter and P_convex hull_ corresponds to the perimeter of the convex hull. Statistical differences between conditions were assessed on biological-replicate summaries using the Kruskal–Wallis test followed by Dunn’s multiple comparisons test with Holm correction for HepG2, while IM-9 data were analyzed using the Mann–Whitney U test. The results are presented as mean ± SEM, and *p* < 0.05 was considered statistically significant.

## 3. Results and Discussion

### 3.1. Effect of Fixation Time on Membrane Preservation

**Figure 2:**
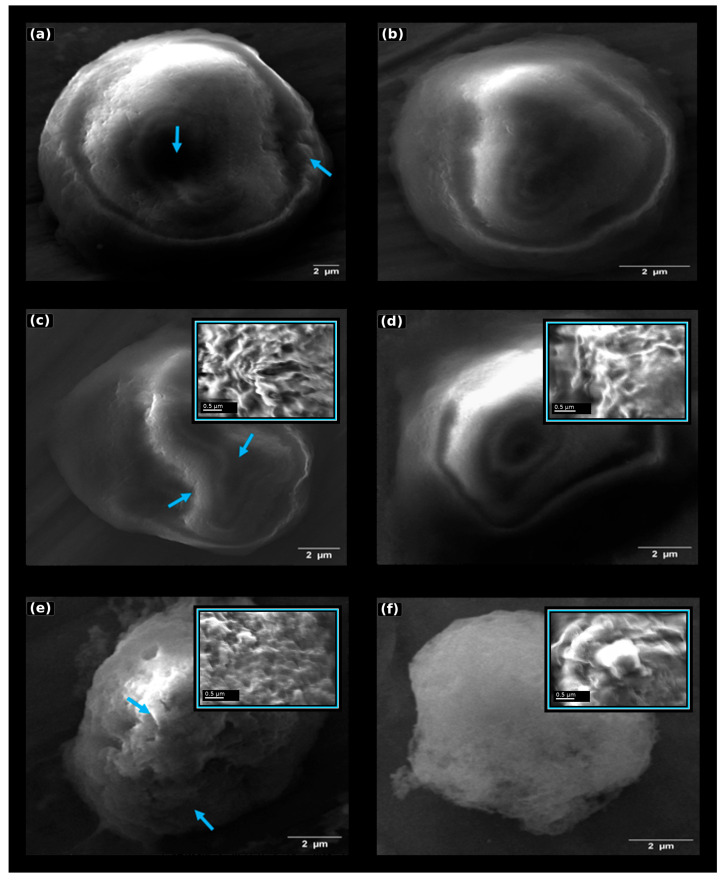
SEM analysis of HepG2 and IM-9 cells under different fixation and drying conditions. (**a**) HepG2 cells fixed for 30 min and air-dried. (**b**) HepG2 cells fixed for 30 min and dried using HMDS (stepwise substitution protocol). (**c**) HepG2 cells fixed for 15 min and air-dried. (**d**) HepG2 cells fixed for 15 min and dried using HMDS (stepwise substitution protocol). (**e**) IM-9 cells fixed for 15 min and air-dried. (**f**) IM-9 cells fixed for 15 min and dried using HMDS (stepwise substitution protocol). Air-dried samples show more evident membrane collapse, surface wrinkling, local flattening, and irregular cell contours, whereas HMDS-treated samples exhibit more preserved cell shape and membrane continuity. Blue arrows indicate representative surface defects, including contour irregularities, wrinkling, and local collapse, in air-dried cells. High-magnification insets in panels (**c**–**f**) show representative local membrane details to better visualize surface texture, folds, and drying-related distortions. Main-panel scale bars: 2 µm; inset scale bars: 0.5 µm. Images are presented to illustrate representative membrane morphology, while quantitative analysis was based on full-cell morphometric measurements.

Two fixation durations were compared to evaluate the effect of fixation time on HepG2 plasma membrane preservation for SEM imaging. In the first experiment, the HepG2 cells (approximately 3.6 × 10^6^ per sample) were fixed in 2% glutaraldehyde for 30 min at 4 °C (Figure 2a,b), whereas in the second experiment the cells were fixed under identical conditions for 15 min (Figure 2c,d) to evaluate the effect of fixation duration on plasma membrane preservation.

The high-resolution insets further emphasize local membrane features that are not fully appreciable in the overview images. These magnified regions were used to support qualitative interpretation of drying-related artefacts, while quantitative comparisons remained based on the full-cell morphometric measurements.

Within the HepG2 conditions tested, reducing fixation time from 30 to 15 min was associated with better structural preservation. SEM imaging indicated that samples fixed for 15 min had better overall membrane preservation, fewer surface artefacts, and a more homogeneous overall morphology. These differences may reflect the chemical effects of glutaraldehyde fixation on membrane stiffness and tolerance to subsequent dehydration and drying steps. Glutaraldehyde reacts primarily with lysine ε-amino groups and other nucleophilic residues, forming reversible Schiff bases that subsequently stabilize into more permanent methylene and bis-alkyl bridges [21,22]. Prolonged exposure increases the probability of polymerisation of glutaraldehyde itself, generating higher-order oligomers that can penetrate the membrane irregularly and introduce structural stiffness [23]. Excessive crosslink density decreases membrane flexibility, increases brittleness, and sensitizes the surface to deformation during dehydration and solvent exchange [23,24]. By limiting the reaction time to 15 min, the fixative establishes sufficient protein crosslinking to stabilize the membrane while minimizing the formation of over-crosslinked or polymerised glutaraldehyde networks. Temperature also contributes to these effects: fixation at 4 °C slows glutaraldehyde polymerisation kinetics and reduces diffusional stress on the membrane, promoting more uniform covalent stabilization [25,26]. In addition to fixation kinetics, the improved preparation conditions contributed to improved ultrastructural stability [21,27]. Controlled processing reduces osmotic fluctuations during buffer washing and ethanol dehydration and limits mechanical stress during centrifugation, thereby improving the consistency of the final SEM morphology [21]. To explore whether the selected 15 min workflow could also be applied to a suspension model, the IM-9 human B-lymphoblastoid cells were processed using the same protocol as the second HepG2 experiment, i.e., 3.6 × 10^6^ cells fixed in 2% glutaraldehyde for 15 min at 4 °C. The SEM analysis showed that this regime preserved overall cell shape and plasma membrane continuity in the IM-9 cells, providing a suitable basis for comparing drying strategies (Figure 2e,f). Because the IM-9 cells were not processed under the 30 min condition, these data support the applicability of the selected workflow to this cell model but do not constitute a full fixation time optimization for suspension cells. Culture medium composition may influence baseline cellular morphology; however, the focus of this study was methodological evaluation under consistent experimental conditions.

### 3.2. Morphometric Analysis of Membrane Preservation

**Figure 3:**
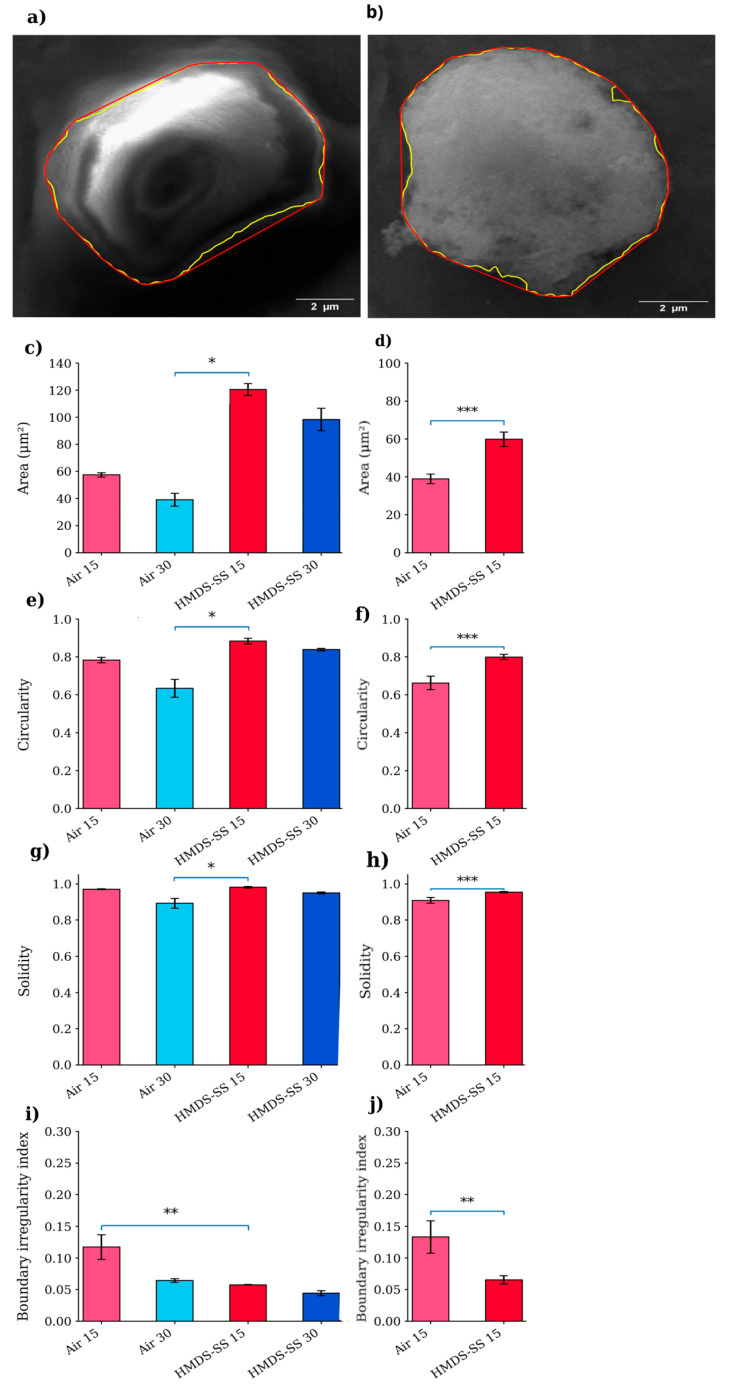
The quantitative morphometric analysis of the HepG2 and IM-9 cells following air-drying and stepwise ethanol-to-HMDS drying. The representative contour extraction and convex hull determination are shown for the HepG2 (**a**) and IM-9 cells (**b**). The yellow and red outlines indicate the cell perimeter and convex hull, respectively. The quantitative analysis of the cell area (**c**,**d**), circularity (**e**,**f**), solidity (**g**,**h**), and boundary irregularity index (**i**,**j**) is shown for the HepG2 (**c**,**e**,**g**,**i**) and IM-9 cells (**d**,**f**,**h**,**j**). The data are shown for 15 cells per condition sampled from four independent biological replicates; statistical testing was performed on biological-replicate summaries as described in Materials and Methods. The statistical significance for HepG2 was assessed using the Kruskal–Wallis test followed by Dunn’s multiple comparisons test with Holm correction, whereas the IM-9 data were analyzed using the Mann–Whitney U test (* *p* < 0.05, ** *p* < 0.01, *** *p* < 0.001). Air 15/30: air-dried (15/30 min); HMDS-SS 15/30: stepwise substitution (EtOH→HMDS, 15/30 min).

To quantitatively assess the observed morphological differences, a morphometric analysis was performed by measuring cell area, circularity, solidity, and boundary irregularity index (BII) (Figure 3). The quantitative results were consistent with the qualitative SEM observations. In the HepG2 cells, significant differences were observed across the tested conditions. In particular, the HMDS-SS 15 condition showed significantly higher area, circularity, and solidity compared with Air 30, while the boundary irregularity index was significantly lower compared with Air 15 (Figure 3c,e,g,i). In the IM-9 cells, stepwise ethanol-to-HMDS drying yielded significantly higher area, circularity, and solidity, together with a lower boundary irregularity index, than air-drying (Figure 3d,f,h,j). Overall, these results indicate that HMDS-based drying is associated with improved morphometric preservation compared with air-drying, particularly under the 15 min fixation condition. Collectively, these findings identify 15 min fixation combined with stepwise ethanol-to-HMDS drying as the most favourable HepG2 condition among those tested and support the same drying trend in IM-9 under the 15 min fixation workflow.

### 3.3. Effect of Drying Strategy on Ultrastructural Preservation

Since SEM requires fully dehydrated samples to avoid artefacts caused by water evaporation under high vacuum conditions, two drying approaches were evaluated: air-drying (Experiment 1) and chemical drying with HMDS (Experiment 2). Chemical drying produced improved morphological preservation (Figure 2b,d,f), with reduced shrinkage and collapse of the membrane structures. In contrast, air-drying resulted in partial deformation and irregular membrane profiles (Figure 2a,c,e). These artefacts included membrane collapse, surface wrinkling, local flattening of the cell body, and the loss of fine protrusions such as microvilli-like structures. These differences arise from the physicochemical properties of the drying agents. Ethanol remaining after dehydration has a high surface tension (~22.3 mN/m at 20 °C), producing strong capillary forces during evaporation that compress and deform soft biological surfaces [28]. HMDS, in contrast, has a much lower surface tension (~18 mN/m at 25 °C) and a substantially higher vapour pressure (~3300 Pa at 25 °C), enabling faster and gentler evaporation [15,18,29,30]. The gradual substitution of ethanol with HMDS through controlled EtOH:HMDS ratios reduces interfacial stress and minimizes the capillary collapse of the membrane [19,30]. HMDS forms a volatile film that evaporates without generating significant pulling forces, allowing the nanoscale features of the plasma membrane to remain intact [28,30]. Overall, the combination of 15 min fixation and HMDS-mediated drying yielded the best-preserved SEM morphology among the tested conditions. The improved membrane preservation observed with stepwise ethanol-to-HMDS substitution reflects reduced capillary forces and surface tension during solvent exchange, while gradual solvent replacement limits osmotic and mechanical stress on the cell cortex, thereby maintaining membrane continuity and fine surface features.

### 3.4. Long-Term Stability of Prepared Samples

**Figure 4:**
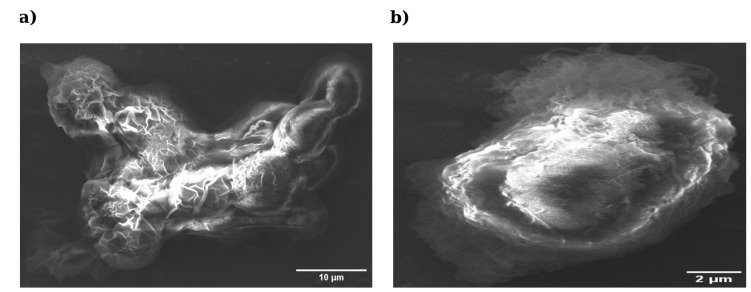
Long-term ultrastructural stability of HepG2 cells after 4 months of storage at 4 °C. (**a**) Cells fixed for 15 min (3.6 × 10^6^ cells) and air-dried (mag = 4.48 k×; scale bar = 10 µm). (**b**) Cells fixed for 15 min (3.6 × 10^6^ cells) and dried using stepwise ethanol-to-HMDS substitution (mag = 9.24 k×; scale bar = 2 µm). Freshly prepared counterparts are shown in Figure 2c,d. Images were acquired at different magnifications and are presented for qualitative illustration only; direct morphometric comparison is not intended.

To assess the robustness of the protocol beyond immediate post-processing morphology, the long-term stability of fixed and dried samples was evaluated after four months of storage at 4 °C (Figure 4). The difference in magnification between panels reflects field selection to best visualize the morphology of each sample. When compared with the corresponding freshly prepared samples shown in Figure 2c,d, the HMDS-dried cells retained a more compact and continuous morphology after 4 months of storage, whereas the air-dried samples appeared markedly collapsed and structurally altered. The HepG2 cells fixed for 15 min and air-dried exhibited marked structural deterioration (Figure 4a), characterized by the collapse of the cell body, surface wrinkling and the loss of membrane definition, indicating that air-dried preparations remain susceptible to delayed capillary-driven deformation. In contrast, the HMDS-processed cells preserved their overall shape, membrane continuity and overall surface integrity even after prolonged storage (Figure 4b). These observations suggest that HMDS may support better structural stability during storage under the tested conditions.

### 3.5. Comparison with Existing SEM Preparation Methods

The comparison summarized in Table 1 highlights methodological differences in fixation and drying strategies commonly used for SEM preparation of mammalian cells. Long glutaraldehyde fixation and air-drying may introduce significant membrane artefacts, whereas CPD and HMDS-based drying are intended to reduce surface-tension-related deformation, although they differ substantially in cost, complexity, safety requirements, and accessibility [6,12,14,15,18]. Critical point drying (CPD) remains a benchmark method because it eliminates surface tension effects during the liquid–gas transition; however, the present study did not include direct CPD or sputter-coated preparations [12,31]. Consequently, the current data cannot establish equivalence or superiority relative to CPD/sputter-coated SEM workflows, and all conclusions should be limited to the tested fixation and drying conditions. The workflow evaluated here integrates short glutaraldehyde fixation with controlled HMDS-mediated drying, avoiding osmium tetroxide (OsO_4_) post-fixation and specialized CPD instrumentation. In the IM-9 cells, only the selected 15 min fixation workflow was assessed; therefore, these results indicate preliminary applicability to a suspension model and support the comparison of drying strategies, not fixation time optimization across suspension cells. The IM-9 samples demonstrated the same qualitative drying-related trends observed in HepG2: air-dried preparations exhibited membrane collapse and surface wrinkling, whereas HMDS-processed cells retained spherical morphology and better-preserved surface features. Together, these data support a simple, low-cost, and reproducible HMDS-based preparation strategy for the two cell models and conditions examined here.

## 4. Conclusions

In this study, we developed and evaluated an improved workflow for scanning electron microscopy (SEM) preparation of cultured cells, focusing on the preservation of plasma membrane ultrastructure. By evaluating fixation duration and drying strategy, we demonstrated that a shorter glutaraldehyde fixation time (15 min) combined with stepwise ethanol-to-HMDS substitution provides improved morphological preservation compared with longer fixation and air-drying approaches. The evaluated protocol resulted in improved membrane continuity, reduced surface artefacts, and enhanced structural stability, as confirmed by both qualitative SEM observations and quantitative morphometric analysis. Importantly, the workflow was successfully applied to both adherent (HepG2) and suspension (IM-9) cell models, suggesting its applicability across different cellular architectures. The proposed approach represents a simple, cost-effective, and reproducible alternative to preparation methods that require specialized equipment such as critical point drying systems. In addition, the improved preservation of membrane features and long-term sample stability highlights its potential utility in studies where the accurate visualization of cell surface morphology is essential. Overall, this workflow represents a practical and accessible strategy for high-quality SEM imaging of cultured cells and may facilitate more reliable morphological analyses in cell biology, bioengineering, and related fields.

## 5. Limitations

This protocol uses chemical fixation and dehydration which may introduce artefacts if the washing and transition steps are not performed gently; care should be taken to minimize mechanical stress during cell detachment, pelleting, and solvent exchange.

A further limitation is that long-term storage stability was assessed qualitatively and using images acquired at different magnifications.

The study did not include a direct comparison with critical point drying (CPD) or sputter-coated preparations due to equipment limitations; therefore, the conclusions are limited to the tested workflow and should not be interpreted as evidence of equivalence or superiority relative to CPD/sputter-coated SEM preparation. Future studies including direct CPD and conductive coating comparisons would be valuable to benchmark ultrastructural fidelity and resolution. In addition, the HepG2 cells were cultured in RPMI-1640 rather than the ATCC-recommended EMEM formulation to maintain a consistent medium background across the workflow; this deviation may influence baseline morphology and should be considered when comparing the present results with standard HepG2 culture protocols.

HMDS is volatile and potentially hazardous; all steps involving HMDS should be performed in a chemical fume hood with appropriate personal protective equipment.

Although the workflow was applied to HepG2 (adherent) and IM-9 (suspension) cells, the IM-9 cells were evaluated only under the selected 15 min fixation condition; therefore, the IM-9 results support preliminary applicability of the workflow to a suspension model but do not validate fixation time optimization across suspension cell conditions. Centrifugation speed and pellet handling may also need optimization depending on cell type, size, and fragility.

SEM imaging quality also depends on coating and microscope settings; in this study the samples were imaged without conductive coating, but users with access to sputter coating may optimize coating thickness according to their instrument and specimen.

## Figures and Tables

**Table 1:** Comparison of selected fixation and drying strategies used for SEM preparation of cultured cells.

Method	Key Characteristics	Advantages	Disadvantages
Long glutaraldehyde fixation (≥30 min) [12]	2–2.5% GA; 30–60 min; OsO_4_ post-fixation	Strong crosslinking; stable samples	Over-fixation; membrane stiffening; OsO_4_ toxicity
Paraformaldehyde fixation (long, low strength) [14]	4% PFA; 24 h at 4 °C; no OsO_4_ post-fixation	Gentle fixation; suitable for morphometrics	Poor membrane preservation; dehydration artefacts
Air-drying after ethanol series [18]	Ethanol dehydration → direct evaporation	Simple; fast; no equipment required	High surface tension → collapse, wrinkling, shrinkage
HMDS chemical drying [15,18]	Common HMDS drying approach: direct replacement of absolute ethanol with HMDS	Very good membrane preservation	HMDS toxicity/flammability; requires fume hood
Critical point drying (CPD) [6]	CO_2_ liquid–gas transition eliminates surface tension	Gold standard for ultrastructure	Expensive; slow; specialized equipment
Evaluated workflow (this study)	2% GA, 15 min; no OsO_4_; stepwise EtOH-to-HMDS; slow HMDS evaporation	Best preservation among tested conditions; low cost; no specialized hardware	No CPD/sputter benchmark; HMDS safety considerations

## Data Availability

The data presented in this study are available on request.
